# Supplementary material for: Impact of Pre-Analytical and Analytical Variables Associated with Sample Preparation on Flow Cytometric Stainings Obtained with EuroFlow Panels
Source: Cancers (Basel). 2022 Jan 18;14(3):473. doi: 10.3390/cancers14030473 (PMC8833630; doi:10.3390/cancers14030473)
Supplement: Supplementary file 1 [file cancers-14-00473-s001.zip › cancers-1547380-supplementary.pdf]

Supplementary materials

S1. Sample preparation protocol for evaluation of OneFlow kits (BD)

Sample preparation:

1. Pipette 300µL of the sample into a 15mL conical tube
2. Add 10mL PBS + 0.5% BSA + 0.1% NaN_3. Vortex for 30 seconds.
3. Centrifuge for 5 minutes at 540g.
4. Remove as much supernatant as possible using a Pasteur pipette or vacuum system.
5. Resuspend the cell pellet before adding more wash buffer
6. Repeat steps 2-5 two more times for a total of 3 washes.
7. Resuspend the cell pellet in 100µL of PBS + 0.5% BSA + 0.1% NaN_3.

Staining Protocol for the LST tube:

1. Add 100µL of washed blood to the dry OneFlow LST tube. Vortex for 3 seconds.
2. Incubate for 30 minutes at room temperature (RT), protected from light.
3. Add 2mL of 1x FACS Lysing Solution. Vortex for 3 seconds.
4. Incubate for 10 minutes at RT protected from light.
5. Centrifuge for 5 minutes at 540g.
6. Discard the supernatant using a Pasteur pipette or vacuum system without disturbing the cell pellet.
7. Add 2mL of PBS + 0.5% BSA + 0.1% NaN_3 to the cell pellet. Vortex for 3 seconds.
8. Centrifuge for 5 minutes at 540 g.
9. Discard the supernatant using a Pasteur pipette or vacuum system without disturbing the cell pellet.
10. Resuspend the cell pellet in 200µL PBS + 0.5% BSA + 0.1% NaN_3.
11. Acquire stained samples at the times set based on the specific experimental schedules.

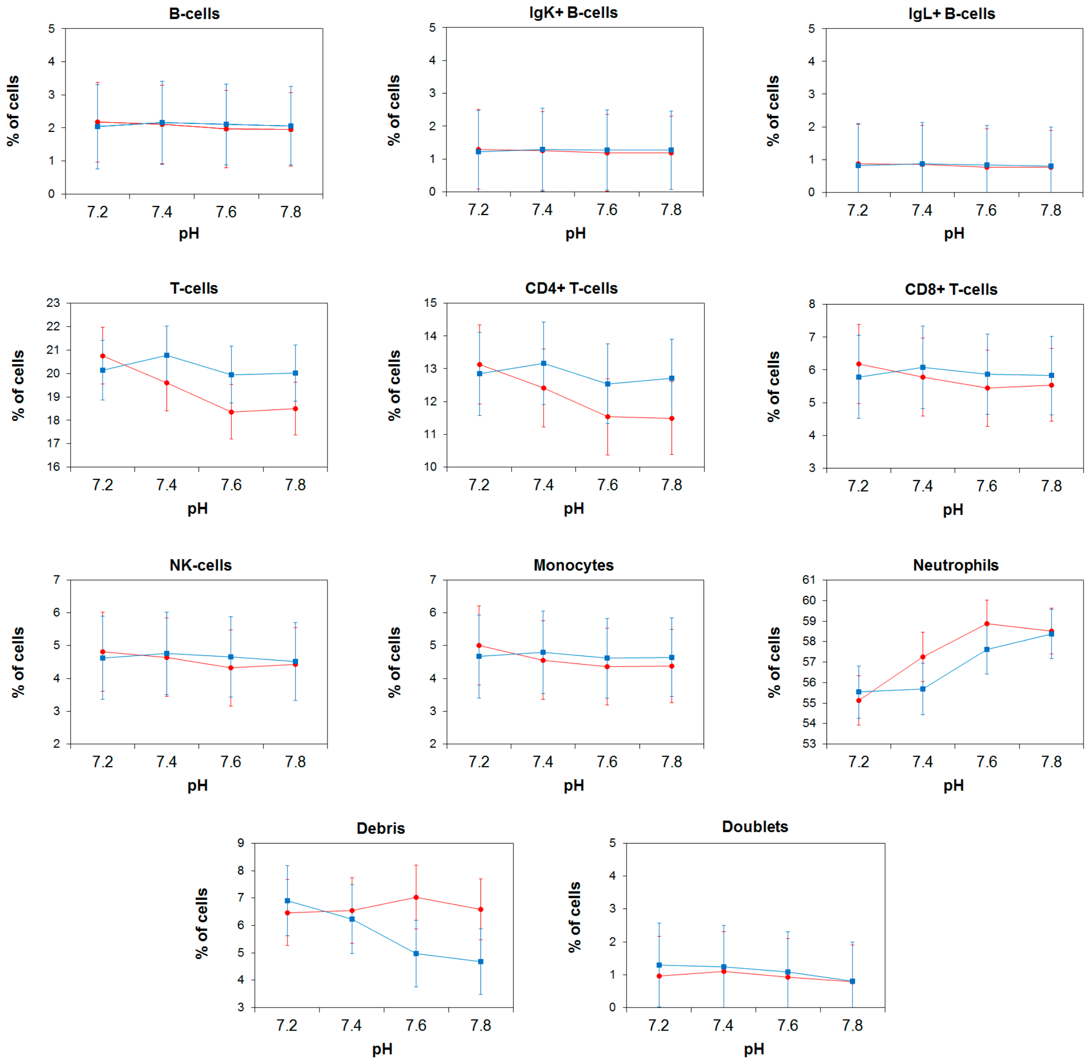

Supplementary Figure S1. Impact of different BSA concentrations (0.2% - red lines vs 0.5% - blue lines) and different pH of the washing buffer on the relative percentage distribution of different cell populations identifiable in normal PB samples with LST (n=20). The whiskers span between ±1SD value of the mean values.

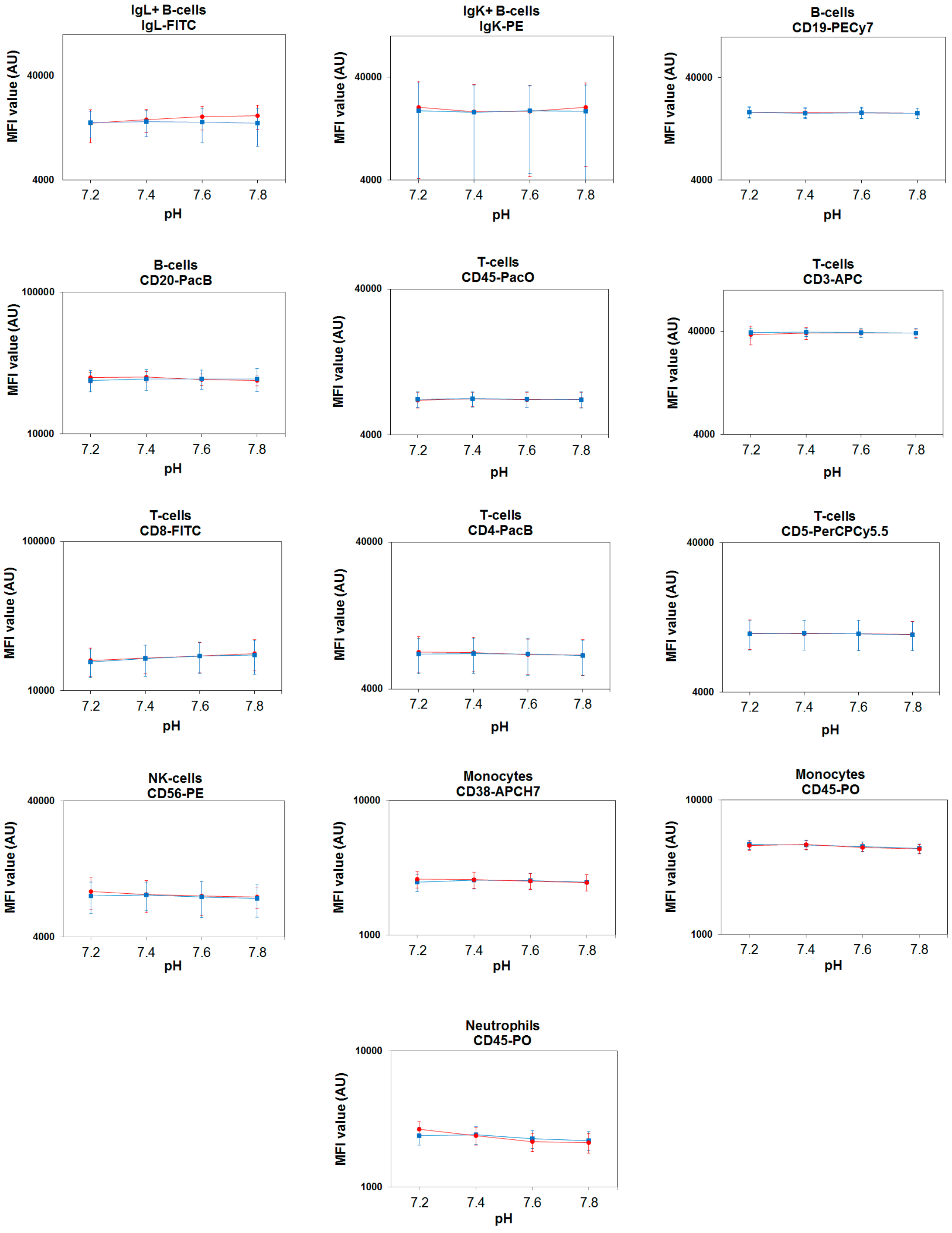

Supplementary Figure S2. Impact of different BSA concentrations (0.2% - red lines vs 0.5% - blue lines) and different pH of the washing buffer on the expression levels of individual LST markers as assessed on different cell populations in normal PB samples (n=20). The whiskers span between ±1SD value of the mean values. APC – allophycocyanin, APC-H7 – allophycocyanin-Hilite 7, FITC – fluorescein isothiocyanate, PacB – Pacific Blue, PE – phycoerythrin, PECy7 – phycoerythrin-cyanine 7, PerCPCy5.5 – peridinin-chlorophyll-cyanine-5.5, PO – Pacific Orange

Supplementary Table S1. Detailed information on the antibody reagents used in the current study, including the antibody titer used per test.

Marker	Fluorochrome	Manufacturer	Clone	Catalogue reference	Staining volume (µl/test)	EuroFlow panel (J. J.M. Van Dongen et al. 2012)
CD2	PacB	Biolegend	TS1/8	309216	1	NK-CLPD
CD2	PECy7	BD	S5.2	335821	2	T-CLPD
CD3	APC	BD	SK7	345767	2.5	LST, B-CLPD
CD3	APCH7	BD	SK7	641415	3	ALOT
CD3	PacB	BD	UCHT1	558117	7	ALOT (cy)
CD3	PerCPCy5.5	BD	SK7	332771	10	T-CLPD, NK-CLPD
CD4	PacB	Biolegend	RPA-T4	300521	0.5	LST, B-CLPD, T-CLPD
CD5	APC	BD	L17F12	345783	2.5	NK-CLPD
CD5	FITC	BD	L17F12	345781	10	T-CLPD
CD5	PerCPCy5.5	BD	L17F12	341109	15	LST, B-CLPD
CD7	APC	eBioscience	124-1D1	17-0079-42	2	ALOT
CD7	FITC	BD	4H9	347483	10	T-CLPD, NK-CLPD
CD8	APCH7	BD	SK1	641400	5	T-CLPD
CD8	FITC	Cytognos	UCHT4	CYT-SLPC-50	20 (part of LST)	LST, B-CLPD
CD9	PacB	Exbio	MEM-61	PB-208-T100	4	BCP-ALL
CD10	APC	BD	HI10A	332777	5	BCP-ALL
CD10	PE	Beckman Coulter	ALB1	A07760	20	B-CLPD
CD10	APCH7	BD	HI10a	EU: 655404 US: 655426	5	AML
CD11b	APC	BD	D12	333143	5	AML
CD11c	APC	BD	S-HCL-3	333144	2	T-CLPD, NK-CLPD
CD11c	PerCPCy5.5	BD	B-Ly6	658330	10	B-CLPD
CD13	PE	BD	L138	347406	7	BCP-ALL, AML
CD15	FITC	BD	MMA	332778	10	BCP-ALL
CD16	PacB	Biolegend	3G8	302032	5	NK-CLPD
CD16	PECy7	BD	3G8	557744	2	T-CLPD
CD19	APCH7	BD	SJ25C1	641395	5	NK-CLPD
CD19	PECy7	Beckman Coulter	J3-119	IM3628	5	ALOT, LST, BCP-ALL, PCD, B-CLPD
CD20	PacB	Biolegend	2H7	302320	1	LST, BCP-ALL, B-CLPD
CD21	PacB	Exbio	LT21	PB-306-T100	4	BCP-ALL
CD22	APC	BD	S-HCL-1	333145	5	BCP-ALL
CD22	PerCPCy5.5	BD	S-HCL-1	658329	25	B-CLPD
CD23	FITC	Dako	MHM6	F7062	2.5	B-CLPD
CD24	APCH7	BD	ML5	EU: 658331	5	BCP-ALL
CD25	PE	BD	2A3	341011	10	T-CLPD, NK-

						CLPD
CD26	PE	BD	L272	340423	10	T-CLPD, NK-CLPD
CD27	APC	BD	L128	337169	2.5	B-CLPD
CD27	FITC	BD	L128	340424	10	T-CLPD
CD27	PerCPCy5.5	BD	L128	EU: 656643 US: 655429	20	PCD
CD28	APC	BD	CD28.2	559770	10	T-CLPD
CD28	PE	BD	L293	348047	20	PCD
CD30	PE	BD	BerH8	550041	10	T-CLPD
CD31	FITC	BD	WM59	555445	10	B-CLPD
CD33	PE	BD	P67.6	345799	5	BCP-ALL
CD34	PerCPCy5.5	BD	8G12	347222	10	ALOT, BCP-ALL, AML
CD38	APCH7	BD	HB7	EU: 656646 US: 653314	3	LST, BCP-ALL, B-CLPD
CD38	FITC	Cytognos	LD38	CYT-38F	3*	PCD
CD38	pure	Cytognos	LD38	CYT-38P1	2*	PCD
CD39	PE	BD	TÜ66	555464	10	B-CLPD
CD43	APCH7	BD	IG10	EU: 655407 US: 655430	2.5	B-CLPD
CD45	PacB	Dako	T29/33	PB986	5	PCD
CD45	PacO	Invitrogen	HI30	MHCD4530	5	ALOT, LST, BCP-ALL, AML, B-CLPD, T-CLPD, NK-CLPD
CD45RA	APC	BD	HI100	550855	10	T-CLPD
CD45RO	PECy7	BD	UCHL1	337168	2	T-CLPD
CD49d	APCH7	BD	9F10	EU: 658332	1	B-CLPD
CD56	PE	Cytognos	C5.9	CYT-56PE	5	PCD
CD56	PE	Cytognos	C5.9	CYT-SLPC-50	20 (Part of LST)	LST, B-CLPD
CD56	PECy7	Beckman Coulter	N901	A21692	5	NK-CLPD, T-ALL
CD57	FITC	BD	HNK-1	333169	10	T-CLPD, NK-CLPD
CD58	FITC	BD	1C3	555920	7	BCP-ALL
CD62L	FITC	BD	SK11	347443	2.5	B-CLPD
CD65	FITC	Beckman Coulter	88H7	IM1654U	7	BCP-ALL
CD66c	PE	Beckman Coulter	KOR-SA3544	IM2357U	10	BCP-ALL
CD79a	PE	Dako	HM57	R7159	5	ALOT (cy)
CD79b	PerCPCy5.5	BD	SN8	656644	10	B-CLPD
CD81	APCH7	BD	JS-81	EU: 655410 US: 655434	5	PCD, BCP-ALL, B-CLPD
CD94	APC	BD	HP-3D9	559876	5	T-CLPD, NK-

						CLPD
CD95	PE	BD	DX2	555674	20	B-CLPD
CD103	FITC	BD	Ber-ACT8	333155	2	B-CLPD
CD117	PECy7	Beckman Coulter	104D2D1	EU: B49221 US: IM3698	5	AML
CD117	APC	BD	104D2	333233	5	PCD, BCP-ALL
CD123	APC	Miltenyi Biotec	AC145	130-090-901	7	BCP-ALL
CD138	PacO	Exbio	B-A38	PO-520	4	PCD
CD185	APC	R&D Systems	51505	FAB190A	10	B-CLPD
CD197	PE	eBioscience	3D12	12-1979	10	T-CLPD
CD200	APC	eBioscience	OX104	17-9200	1.25	B-CLPD
CD279	PE	BD	MIH4	557946	20	T-CLPD
CD305	PE	BD	DX26	550811	10	B-CLPD
β2micro	PerCPCy5.5	BD	Tü99	EU: 656645 US: 655435	15	PCD
Granzyme B	PE	Sanquin	CLB-GB11	M2289	15	T-CLPD (cy), NK-CLPD (cy)
HLADR	PacB	BioLegend	L243	307624	1 (1:5 dilution)	AML, NK-CLPD
HLADR	PECy7	BD	L243	335830	2.5	T-CLPD
HLADR	PerCPCy5.5	BD	L243	552764	10	B-CLPD
Igκ	APC	Dako	polyclonal rabbit serum	C0222	2.5	PCD (cy)
Igκ	PacB	Exbio	A8B5	PB-504-T100	4	BCP-ALL
Igκ	PE	Cytognos	polyclonal	CYT-SLPC-50	20 (Part of LST)	LST, B-CLPD
Igλ	APCC750	Cytognos	polyclonal	CYT-LAC750	3	PCD (cy), BCP-ALL
Igλ	FITC	Cytognos	polyclonal	CYT-SLPC-50	20 (Part of LST)	LST, B-CLPD
Igμ	FITC	Dako	polyclonal rabbit serum	F0058	10	BCP-ALL (cy)
IgM	APC	BD	G20-127	551062	10	BCP-ALL, B-CLPD
cyMPO	FITC	Dako	MPO-7	F0714	3	ALOT (cy)
NG2	PE	Beckman Coulter	7.1	IM3454U	10	BCP-ALL, AML
Perforin	FITC	BD	δG6	556577	10	T-CLPD (cy), NK-CLPD (cy)
TCL1	APC	eBioscience	eBio1-21	17-6699	2	T-CLPD (cy)
TCRγδ	PECy7	BD	11F2	EU: 655410 US: 655434	3	LST, B-CLPD
nuTdT	FITC	Dako	HT-6	F7139	10	BCP-ALL (nu)

APC – allophycocyanin, APC-H7 – allophycocyanin-Hilite 7, APCC750 – allophycocyanin-cyanine 750, FITC – fluorescein isothiocyanate, MPO – myeloperoxidase, PacB – Pacific Blue, PE – phycoerythrin, PECy7 – phycoerythrin-cyanine 7, PerCPCy5.5 – peridinin-chlorophyll-cyanine-5.5, PacO – Pacific Orange, TdT – terminal nucleotide transferase, cy – cytoplasmic antigen, nu –

nuclear antigen, * a mixture of fluorochrome-conjugated and unconjugated antibodies was used to reduce the signal intensity, while retaining saturation conditions in order to avoid unpredictable variation in staining patterns

Supplementary Table S2. Absolute and relative percentage changes of particular cell populations, debris and doublets detectable in BM samples stained with ALOT tube at day 0 and after 24-hour storage.

Case ID	% at day 0	% at +24h	% difference	Normalized % difference
Debris				
LO-2568	2.1	2.9	0.8	+38
LU-2584	1.2	2.9	1.7	+142
MON-0139	0.2	0.2	0	0
PO-2570	0.6	0.3	-0.3	-50
RZ-2580	4.8	2.5	-2.3	-48
WR-2567	2.3	3.2	0.9	+39
WR-2569	0.9	1.1	0.2	+22
Mean ± SD [%]	1.7 ± 1.6	1.9 ± 1.3	N/A	
Mean increase [%]<br>Mean decrease [%]	N/A		0.7<br>-1.3	+60<br>-49
Cell doublets				
LO-2568	2.3	2.6	0.3	+13
LU-2584	2.6	15.9	13.3	+512
MON-0139	3.6	3.3	-0.3	-8
PO-2570	13.1	20.7	7.6	+58
RZ-2580	3.4	4.9	1.5	+44
WR-2567	6.2	3.5	-2.7	-44
WR-2569	5.6	4.1	-1.5	-27
Mean ± SD [%]	5.3 ± 3.8	7.9 ± 7.3	N/A	
Mean increase [%]<br>Mean decrease [%]	N/A		3.1<br>-1.5	+51<br>-35
Unspecific staining with APC-H7 fluorochrome				
LO-2568	0.5	1.2	0.7	+140
LU-2584	1.0	4.2	3.2	+320

MON-0139	0.3	0.4	0.1	+33
PO-2570	0.1	6.9	6.8	+6800
RZ-2580	0.6	13.4	12.8	+2133
WR-2567	0.8	1.6	0.8	+100
WR-2569	0.1	0.1	0	0
Mean ± SD [%]	0.5 ± 0.3	4.0 ± 4.8	N/A	
Mean increase [%]<br>Mean decrease [%]	N/A		1.2<br>N/A	+148<br>N/A
Blasts				
LO-2568	85.4	84.1	-1.3	-2
LU-2584	76.7	63.2	-13.5	-18
MON-0139	88.1	88.3	0.2	0
PO-2570	82.9	75.2	-7.7	-9
RZ-2580	77.9	76.6	-1.3	-2
WR-2567	80.7	85.1	4.4	+5
WR-2569	85.8	88.4	2.6	+3
Mean ± SD [%]	83.5 ± 3.8	83.0 ±5.7	N/A	
Mean increase [%]<br>Mean decrease [%]	N/A		2.4<br>-3.4	N/A<br>N/A
Neutrophils				
LO-2568	0.8	0.8	0	0
LU-2584	3.7	3.8	0.1	+3
MON-0139	0.4	0.4	0	0
PO-2570	2.4	2.8	0.4	+17
RZ-2580	4.3	5.6	1.3	+30
WR-2567	0.2	0.3	0.1	+50
WR-2569	0.6	0.6	0	0
Mean ± SD [%]	1.8 ± 1.7	2.0 ±2.0	N/A	
Mean increase [%]<br>Mean decrease [%]	N/A		0.3<br>N/A	+30<br>N/A
B-cells				
LO-2568	1.7	1.7	0	0
LU-2584	2.1	1.6	-0.5	-24
MON-0139	1.2	1.3	0.1	+8
PO-2570	0.1	0.1	0	0

RZ-2580	2.7	2.2	-0.5	-19
WR-2567	1.7	0.6	-1.1	-65
WR-2569	1.7	1.2	-0.5	-29
Mean ± SD [%]	1.6 ± 0.8	1.2 ±0.7	N/A	
Mean increase [%]<br>Mean decrease [%]	N/A		0.1<br>-0.5	N/A<br>-34
T-cells				
LO-2568	6.3	6.5	0.2	+3
LU-2584	8.3	7.2	-1.1	-13
MON-0139	5.7	5.8	0.1	+2
PO-2570	0.8	0.9	0.1	+13
RZ-2580	4.3	4.9	0.6	+14
WR-2567	8.3	6.3	-2.0	-24
WR-2569	3.2	3.0	-0.2	-6
Mean ± SD [%]	5.3 ± 2.0	4.9 ±2.2	N/A	
Mean increase [%]<br>Mean decrease [%]	N/A		0.2<br>-1.1	N/A<br>-24
NK-cells				
LO-2568	0.8	0.8	0	0
LU-2584	2.6	2.5	-0.1	-4
MON-0139	0.6	0.5	-0.1	-17
PO-2570	0.2	0.1	-0.1	-50
RZ-2580	0.5	0.5	0	0
WR-2567	0.4	0.3	-0.1	-25
WR-2569	0.9	0.9	0	0
Mean ± SD [%]	0.9 ± 0.8	0.8 ±1.1	N/A	
Mean increase [%]<br>Mean decrease [%]	N/A		N/A<br>-0.1	N/A<br>-37

N/A – not applicable; normalized % difference calculated as [(+24h-day 0)/(day 0)]x100 [%]

Supplementary Table S3. Ratios of percentage of debris, doublets and pathological/clonal B-cells in particular BM and PB samples stained with LST tube at different time points.

Case ID	% at 0h	% at 3h	% at 24h	% at (24h+3h)	% difference 3h vs 0h	% difference 24h vs 0h	% difference (24h+3h) vs 24h	Normalized % difference 3h vs 0h	Normalized % difference 24h vs 0h	Normalized % difference (24h+3h) vs 24h
Debris										
34343	3.6	3.9	1.5	1.5	0.3	-2.1	0	+8	-58	0
34344	0.2	0.2	0.7	3.7	0	0.5	3.0	0	+250	+429
34345	9.5	8.5	11.7	8.6	-1.0	2.2	-3.1	-11	+23	-26
34346	0.3	0.2	0.6	0.3	-0.1	0.3	-0.3	-33	+100	-50
34419	3.1	1.4	5.5	4.6	-1.7	2.4	-0.9	-55	+77	-16
34420	0.8	0.4	1.6	1.7	-0.4	0.8	0.1	-50	+100	+6
34457	1.1	1.4	2.9	1.8	0.3	1.8	-1.1	+27	+164	-38
001	0.7	0.6	3.8	3.5	-0.1	3.1	-0.3	-14	+443	-8
002	0.7	N/A	0.7	N/A		0			0	
003	1.7	N/A	1.5	N/A		-0.2			-12	
004	0.1	N/A	0.1	N/A		0			0	
005	4.4	N/A	3.9	N/A		-0.5			-11	
006	0.6	0.6	N/A	N/A	0			0		
Mean ± SD [%]	2.0 ± 2.6	1.9 ± 2.7	2.9 ± 3.2	3.2 ± 2.6	N/A					

Mean increase [%]<br>Mean decrease [%]	N/A				0.3<br>-0.7	1.6<br>-0.9	1.6<br>-1.1	+18<br>-33	+165<br>-27	+217<br>-28
Cell doublets										
34343	3.0	2.7	2.8	2.3	-0.3	-0.2	-0.5	-10	-7	-18
34344	7.3	5.8	8.3	6.2	-1.5	1.0	-2.1	-21	+14	-25
34345	2.1	2.4	2.0	2.7	0.3	-0.1	0.7	+14	-5	+35
34346	7.0	6.5	8.2	7.8	-0.5	1.2	-0.4	-7	+17	-6
34419	1.7	1.1	2.1	1.7	-0.6	0.4	-0.4	-35	+24	-19
34420	1.3	1.5	2.2	2.7	0.2	0.9	0.5	+15	+69	+23
34457	2.4	2.5	2.3	2.6	0.1	-0.1	0.3	+4	-4	+13
001	4.7	5.1	3.0	5.4	0.4	-1.7	2.4	+9	-36	+80
002	1.9	N/A	1.8	N/A		-0.1			-5	
003	8.0	N/A	2.5	N/A		-5.5			-69	
004	5.4	N/A	4.6	N/A		-0.8			-15	
005	4.3	N/A	2.9	N/A		-1.4			-33	
006	5.0	4.1	N/A	N/A	-0.9			-18		
Mean ± SD [%]	4.2 ± 2.3	3.5 ± 1.9	3.6 ± 2.3	4.0 ± 2.2	N/A					
Mean increase [%]<br>Mean decrease [%]	N/A				0.3<br>-0.7	0.9<br>-1.2	1.0<br>-0.9	+11<br>-18	+31<br>-22	+38<br>-41

Pathological / clonal B-cells										
34343	6.9	6.9	5.4	6.0	0.0	-1.5	0.6	0	-22	+12
34344	84.8	86.7	84.7	83.7	1.9	-0.1	-1.0	+2	0	-1
34345	17.8	18.3	19.0	19.8	0.5	1.2	0.8	+3	+7	+4
34346	86.4	86.9	85.4	85.7	0.5	-1.0	0.3	+1	-1	0
34419	3.4	4.0	3.8	4.1	0.6	0.4	0.3	+18	+12	+10
34420	40.6	43.4	44.6	45.6	2.8	4.0	1.0	+7	+10	+2
34457	24.2	24.0	23.3	23.3	-0.2	-0.9	0	-1	-4	0
001	72.1	72.1	50.9	50.6	0.0	-21.2	-0.3	0	-29	-1
002	70.0	N/A	67.7	N/A		-2.3			-3	
003	81.2	N/A	81.8	N/A		0.6			+1	
004	83.4	N/A	79.6	N/A		-3.8			-5	
005	56.7	N/A	62.4	N/A		5.7			+10	
006	81.5	82.3	N/A	N/A	0.8			+1		
Mean ± SD [%]	54.5 ± 31.8	47.2 ± 35.1	50.7 ± 31.1	39.9 ± 32.2	N/A					
Mean increase [%]<br>Mean decrease [%]	N/A				1.0<br>-0.2	2.4<br>-4.4	0.6<br>-0.6	+5<br>-1	+8<br>-11	+6<br>-1

N/A – not applicable; normalized % difference 3h vs 0h calculated as [(3h-0h)/0h]x100 [%]; normalized % difference 24h vs 0h calculated as [(24h-0h)/0h]x100 [%]; normalized % difference (24h+3h) vs 24h calculated as [((24h+3h)-24h)/24h]x100 [%]

Supplementary Table S4. Ratios of percentage of debris, doublets and pathological/clonal B-cells in particular BM and PB samples stained with the 1st tube of B-CLPD panel at different time points.

Case ID	% at 0h	% at 3h	% at 24h	% at (24h+3h)	% difference 3h vs 0h	% difference 24h vs 0h	% difference (24h+3h) vs 24h	Normalized % difference 3h vs 0h	Normalized % difference 24h vs 0h	Normalized % difference (24h+3h) vs 24h
Debris										
001	9.2	8.1	6.9	7.2	-1.1	-2.3	0.3	-12	-25	+4
003	2.4	N/A	2.8	2.2		0.4	-0.6		+17	-21
005	1.1	0.9	0.8	0.9	-0.2	-0.3	0.1	-18	-27	+13
006	1.3	N/A	1.0	0.9		-0.3	-0.1		-23	-10
007	1.8	N/A	2.1	0.8		0.3	-1.3		+17	-62
008	21.0	1.6	3.8	3.1	-19.4	-17.2	-0.7	-92	-82	-18
Mean ± SD [%]	6.1 ± 7.9	3.5 ± 4.0	2.9 ± 2.3	2.5 ± 2.5	N/A					
Mean increase [%]<br>Mean decrease [%]	N/A				N/A<br>-7.0	0.4<br>-5.0	0.2<br>-0.7	N/A<br>-41	+17<br>-39	+8<br>-28
Cell doublets										
001	5.3	4.0	2.8	3.1	-1.3	-2.5	0.3	-25	-47	+11
003	4.7	N/A	5.3	7.3		0.6	2.0		+13	+38
005	7.1	4.3	3.7	2.8	-2.8	-3.4	-0.9	-39	-48	-24

006	4.8	N/A	3.9	2.4		-0.9	-1.5		-19	-38
007	3.3	N/A	7.6	5.7		4.3	-1.9		+130	-25
008	7.8	4.9	16.6	8.9	-3.0	8.8	-7.7	-37	+113	-46
Mean ± SD [%]	5.5 ± 1.7	4.4 ± 0.5	6.7 ± 5.2	5.0 ± 2.7	N/A					
Mean increase [%]<br>Mean decrease [%]	N/A				N/A<br>-2.3	4.6<br>-2.3	1.2<br>-3.0	N/A<br>-34	+85<br>-38	+24<br>-34
Pathological / clonal B-cells										
001	13.1	13.4	13.1	13.9	0.3	0	0.8	+2	0	+6
003	75.4	78.1	71.3	76.8	2.7	-4.1	5.5	+4	-5	+8
005	70.7	N/A	83.1	75.3		12.4	-7.8		+18	-9
006	77.2	N/A	74.6	78.3		-2.6	3.7		-3	+5
007	27.2	N/A	31.7	29.3		4.5	-2.4		+17	-8
008	52.1	81.3	44.2	71.2	29.2	-7.9	27.0	+56	-15	+61
Mean ± SD [%]	52.6 ± 27.0	57.6 ± 38.3	53.0 ± 27.7	57.5 ± 28.3	N/A					
Mean increase [%]<br>Mean decrease [%]	N/A				10.7<br>N/A	8.5<br>-4.9	9.3<br>-5.1	+21<br>N/A	+18<br>-8	+20<br>-9

N/A – not applicable; N/A – not applicable; normalized % difference 3h vs 0h calculated as [(3h-0h)/0h]x100 [%]; normalized % difference 24h vs 0h calculated as [(24h-0h)/0h]x100 [%]; normalized % difference (24h+3h) vs 24h calculated as [((24h+3h)-24h)/24h]x100 [%]

Supplementary Table S5. Ratios of percentage of debris, doublets and pathological/clonal plasma cells in particular BM and PB samples stained with PCD panel at different time points.

Case ID	% at 0h	% at 3h	% at 24h	% at (24h+3h)	% difference 3h vs 0h	% difference 24h vs 0h	% difference (24h+3h) vs 24h	Normalized % difference 3h vs 0h	Normalized % difference 24h vs 0h	Normalized % difference (24h+3h) vs 24h
Debris										
BM1	3.1	2.9	7.2	4.3	-0.2	4.1	-2.9	-6	+132	-40
BM2	3.3	3.1	5.4	4.4	-0.2	2.1	-1.0	-6	+64	-19
BM3	3.4	3.4	6.8	4.9	0	3.4	-1.9	0	+100	-28
BM4	3.6	4.1	8.3	4.8	0.5	4.7	-3.5	+14	+131	-42
BM5	8.9	8.4	12.7	7.6	-0.5	3.8	-5.1	-6	+43	-40
pat 001	0.05	0.05	0.08	0.06	0	0.03	-0.02	0	0	0
pat 005	0.01	N/A	0.06	0.06		0.05	0		0	0
pat 006	28.0†	N/A	37.3†	26.3†		9.3	-11.0		+33	-29
pat 007	2.5	N/A	20.0	2.3		17.5	-17.7		+700	-88
Mean ± SD [%]	3.1 ± 2.8	3.7 ± 2.7	7.6 ± 6.5	3.6 ± 2.6	N/A					

Mean increase [%]<br>Mean decrease [%]	N/A				0.5<br>-0.3	4.6<br>N/A	N/A<br>-4.2	+14<br>-6.0	+172<br>N/A	N/A<br>-41
Cell doublets										
BM1	3.0	3.2	5.0	6.7	0.2	2.0	1.7	+7	+67	+34
BM2	3.5	3.6	6.2	4.3	0.1	2.7	-1.9	+3	+77	-31
BM3	22.7	7.5	9.8	5.7	-15.2	-12.9	-4.1	-67	-57	-42
BM4	4.2	7.9	8.4	10.4	3.7	4.2	2.0	+88	+100	+24
BM5	6.1	8.2	11.2	15.8	2.1	5.1	4.6	+34	+84	+41
pat 001	2.9	2.7	2.3	2	-0.2	-0.6	-0.3	-7	-21	-13
pat 005	4.6		5.2	4.9		0.6	-0.3		+13	-6
pat 006	10.4		2.5	1.9		-7.9	-0.6		-76	-24
pat 007	4.5		6.0	6.4		1.5	0.4		+33	+7
Mean ± SD [%]	4.9 ± 2.4	6.9 ± 6.4	6.3 ± 3.0	6.5 ± 4.3	N/A					
Mean increase [%]<br>Mean decrease [%]	N/A				1.5<br>-0.2	2.7<br>-0.6	2.2<br>-1.4	+33<br>-37	+62<br>-51	+26<br>-23
Pathological / clonal plasma cells										
BM1	0.2	0.2	0.3	0.3	0	0.1	0	-2	+11	+6

BM2	N/A	N/A	N/A	N/A						
BM3	11.7†	14.1†	12.4†	13.4†	2.4	0.7	1.0	+20	+6	+8
BM4	0.9	0.8	0.8	0.8	-0.1	-0.1	0	-14	-14	0
BM5	0.8	0.8	0.7	0.7	0	-0.1	0	+5	-14	+5
pat 001	0.3	0.3	0.3	0.3	0	0	0	-3	-3	+7
pat 005	0.8	N/A	0.7	0.6		-0.1	-0.1		-19	-5
pat 006	0.1	N/A	0.1	0.1		0	0		0	0
pat 007	2.0	N/A	1.4	1.3		-0.6	-0.1		-29	-11
Mean ± SD [%]	0.7 ± 0.7	0.5 ± 0.3	0.6 ± 0.4	0.6 ± 0.4	N/A					
Mean increase [%]<br>Mean decrease [%]	N/A				2.4<br>-0.1	0.7<br>-0.2	1.0<br>-0.1	+21<br>-11	+28<br>-17	+8<br>-11

† outlier results, N/A – not applicable; N/A – not applicable; normalized % difference 3h vs 0h calculated as [(3h-0h)/0h]x100 [%]; normalized % difference 24h vs 0h calculated as [(24h-0h)/0h]x100 [%]; normalized % difference (24h+3h) vs 24h calculated as [((24h+3h)-24h)/24h]x100 [%]

Supplementary Table S6. MFI values of LST markers evaluated on relevant cell populations obtained at different pH and BSA concentrations.

	pH / BSA concentration [%]							
Cell population / marker	7.2 / 0.2	7.2 / 0.5	7.4 / 0.2	7.4 / 0.5	7.6 / 0.2	7.6 / 0.5	7.8 / 0.2	7.8 / 0.5
B-cells								
CD19								
Mean MFI	18307	18415	18188	17918	18145	18018	17894	17954
SD	1996	2196	2162	2021	2339	2084	2153	2100
CD20								
Mean MFI	25082	23910	25292	24411	24161	24351	23860	24368
SD	4187	4104	4265	4081	4732	3756	5174	4400
IgKappa								
Mean MFI	20322	18817	18308	18123	18501	18811	20247	18545
SD	16180	13707	15359	15530	14198	15232	14861	13289
IgLambda								
Mean MFI	14040	14181	15214	14505	16282	14323	16542	14021
SD	4939	4017	3859	4037	4196	5233	4374	5599
T cells								
CD3								
Mean MFI	37391	38892	38472	39413	38672	38847	38547	38408
SD	7681	4222	5098	3769	3606	4107	3537	4076
CD5								
Mean MFI	9916	9815	9852	9865	9837	9796	9758	9685
SD	2213	2193	2227	2225	2261	2223	2162	2112
CD4								
Mean MFI	7111	6941	7105	6996	6859	6899	6795	6759
SD	1933	1857	1886	1873	1851	1935	1849	1852
CD8								
Mean MFI	15895	15585	16579	16369	17092	17068	17690	17367
SD	3389	3341	3643	3856	3881	4006	4159	4460
CD45								
Mean MFI	6970	7068	7101	7101	7035	7075	7064	7030
SD	837	869	804	866	842	890	835	891
NK cells								
Mean MFI	8603	7970	8172	8135	7956	7857	7837	7664
SD	2307	2069	2172	1903	2238	2329	1418	2093
Monocytes								
CD38								
Mean MFI	2584	2459	2562	2549	2505	2533	2451	2456
SD	352	367	354	364	329	341	339	349

Supplementary Table S7. Expression of markers on relevant PB cell populations stained with LST tube at different pH on averaging the results for different BSA concentrations.

	Mean MFI of marker on relevant cell population at				higher MFI at higher pH	lower MFI at higher pH
	pH = 7.2	pH = 7.4	pH = 7.6	pH = 7.8		
B-cells						
Igλ (n=15)	14111	14860	**15303**	15282	1 (7%) [+44%]	0
Igκ (n=10)	**19570**	18216	18656	19396	3 (30%) [+73%]	0
CD19 (n=15)	**18530**	18226	18262	18082	0	0
CD20 (n=15)	24496	**24852**	24256	24114	0	0
T-cells						
CD4 (n=15)	7026	**7051**	6879	6777	0	0
CD8 (n=15)	15740	16474	17080	**17528**	0	0
CD45 (n=15)	7019	**7100**	7055	7047	0	0
CD5 (n=15)	**9866**	9858	9817	9722	0	0
CD3 (n=15)	38142	**38942**	38760	38478	0	0
NK-cells						
CD56 (n=15)	**8286**	8153	7906	7750	0	0
Monocytes (n=15)						
CD38 (n=10)	2522	**2556**	2519	2454	0	0

For each antigen, modal pH values were determined at which the lowest and the highest (i.e., extreme) MFI was observed at every pH condition. Thus, each antigen was assigned 2 pH values at which the minimal and maximal MFI values were observed. Results expressed as number (percentage) of cases with significant (i.e., beyond the tolerance range of ±30%) MFI ratio between extreme pH values. Percentages in square brackets indicate mean relative percentage change in MFI; n indicates number of cases positive/evaluated for given antigen. The maximal mean MFI values at respective pH were marked in bold.

Supplementary Table S8. Selected additional markers included in the EuroFlow leukemia/lymphoma panels that were not directly investigated in the current study and that may be influenced by sample preparation-associated variables.

Marker	Variables with possible influence	References
CD16	The expression level decreases on granulocytes in samples stored for >24h	[28]
CD4<br>CD13<br>CD33<br>CD34<br>CD38<br>CD45<br>CD71<br>CD117	The expression levels vary on different cell types between EDTA- and heparin anticoagulated samples	[31]
CD138	The expression level diminishes on PC in samples stored for >24h at 25ºC; the effect more visible when stored at 4ºC;	[37]
	The expression level on PC is decreased in heparin- vs. EDTA-anticoagulated samples	[30]
CD229	The expression level increases after storage at 25ºC for 72h	[37]

PC - plasma cells
